# Supplementary material for: The impact of skim reading and navigation when reading hyperlinks on the web
Source: PLoS One. 2020 Sep 17;15(9):e0239134. doi: 10.1371/journal.pone.0239134 (PMC7497986; doi:10.1371/journal.pone.0239134)
Supplement: Attachment — Submitted filename: Response to Reviewers.docx [file pone.0239134.s001.docx]

**Reviews and Responses**

Reviewer comments are in **bold** text, responses are in standard text.

**Reviewer #1: This manuscript describes two experiments examining the impact of hyperlinks on eye movement behaviour during skim reading and reading for comprehension. Experiment 1 uses static pages, Experiment 2 uses pages with navigable links.

I thought that the Introduction and Experiment 1 read nicely, and I have only minor comments on those. However, there seem to be some major errors in the results section of Experiment 2. The statistics reported in Table 4 don’t match up to the text in several important respects, and also seem odd in relation to the means reported in Table 3. For example, if we take go-past time, according to Table 4 the only significant interaction is between word type and task type. However, the text (lines 617-626) reports and investigates a significant interaction between word frequency and word type, and makes no mention of an interaction between word type and task type.**

There was an error in the Tables for experiment two, with two interaction terms labelled the wrong way around, thank you for pointing this out. We have amended Table 4 to reflect the results accurately (i.e. a significant interaction of word frequency and word type in go-past time).

**When comparing the means in Table 3 to the statistics in Table 4, it would also seem surprising that what appears numerically to be an enormous interaction between task type and word type for go-past time (difference between word type conditions 1014ms when reading for comprehension and 179ms when skim reading) has a t value of only 2.09. Obviously I appreciate that the log transformation will affect things, but given the other issues this may also be an error. These discrepancies are also present for the other measures in this experiment. In light of these I can’t tell what the results actually were, so I’m not in a position to comment on any of the discussion.**

In the amended Tables, it can be seen that there is no significant interaction between task type and word type, despite the large numerical differences noted by the reviewer. This is due to the extremely high level of variability that can occur within go-past time (for instance the standard deviation in these measures for linked/high frequency words is 4215ms). This is because of the nature of eye movement behaviour captured in this measure for this specific experiment. Often readers won’t make regressions, but when they do these regressions can take multiple seconds or even half a minute given that entire segments of a paragraph can be re-read. We hope the amended results section makes this much clearer and is more interpretable.  **Minor comments
It would be helpful to provide a brief description of hyperlinks and hypertext (I was unfamiliar with the latter term).**

This is now provided within the introduction (line 43 and 61)

**Line 74 – Liu rather than Lui**

This has been amended (line 85).

**Line 170 – I would suggest removing “where” as it creates a garden path**

This has been amended.

**The example stimulus figures seem to be missing, and the numbers of the rest don’t match up with the text.**

Links to the stimuli have been inserted into the manuscript, the original figures could not be submitted due to PloS rules on copyright (as the images include the Wikipedia logo). Figure names have been updated accordingly.

**In both of your experiments you have the reading for comprehension block before the skim reading block. I agree that this is the best approach to take, but it may be worth considering what impact this may have had on your results.**

We agree this could have been considered and we have justified our choice further within the manuscript. We have added consideration of this into the method section of Experiment 1. We also briefly mention a pilot study in which participants indicated that it was difficult to switch from skim reading to more thorough reading for comprehension.

**In the tables it would make things easier if you had some lines or spaces separating the interactions – at the moment it’s difficult to work out which number corresponds to which interaction.**

We agree this could have been clearer and the Table 2 and 6 have been amended to provide further clarity.

**Line 382 – As you state that comprehension accuracy was lower for skim reading than reading for comprehension, it would be helpful to provide some statistics for this in the results section.**

We have deleted this point from the manuscript, in light of this comment and others from reviewers, as we wish to ensure the focus of the Experiments is on the reading process, rather than on the comprehension questions.

**Lines 385-391 – It would be better to point out earlier in this section that this study only looked at reading for comprehension.**

This is now pointed out (line 192, 501).

**In Experiment 2 I presume there is necessarily variability in how many data points were available per participant in each condition. Therefore it would be good to have some sense of how much data there was in total and how this was distributed across conditions.**

We did not include this point in the manuscript for the sake of brevity. The distribution of datapoints for our fixation measures across each condition for each experiment are provided below (i.e. the number of datapoints after taking into account data cleaning, removal of outliers and trials where target word was not fixated). All data are freely available online for further verification.

Experiment 1 –

High/Linked/Comprehension – 541

High/Unlinked/Comprehension – 542

Low/Linked/Comprehension – 591

Low/Unlinked/Comprehension – 568

High/Linked/Skimming – 478

High/Unlinked/Skimming – 362

Low/Linked/Skimming – 517

Low/Unlinked/Skimming – 392

Experiment 2 –

High/Linked/Comprehension – 158

High/Unlinked/Comprehension – 162

Low/Linked/Comprehension – 153

Low/Unlinked/Comprehension – 171

High/Linked/Skimming – 135

High/Unlinked/Skimming – 252

Low/Linked/Skimming – 156

Low/Unlinked/Skimming - 284

**Lines 561-563 – Just for gaze duration? It is reported for all the other measures.**

This is correct, and is now reflected in the text (line 726)

**It’s very difficult to see the figures. I appreciate that they always end up in low resolution for reviews, but it would help if the layout was changed a bit (e.g. for the figures where you have three graphs, they could be displayed larger if placed on top of each other rather than side by side).**

We considered this design change but found the figures were often harder to interpret, due to overlap between data points and error bars. We anticipate the reviewer would be ok with our figures if they could inspect them in their full resolution.  **Reviewer #2: Thanks for the opportunity to assess the manuscript “The Impact of Skim Reading and Navigation when Reading Hyperlinks on the Web”. This is a well though, nicely controlled set of studies which aim to identify how hypertexts, and particularly hyperlinks, are processed by means of eye-tracking indicators. Unfortunately, the strong emphasis on experimental controlled materials came to the expense of limiting the construct validity of the task. As a result, the studies resemble more a traditional reading comprehension tasks than hypertext ones. Let me elaborate on this relevant point in the following.**

**The studies used only word level indicators of eye-movements to describe what they define as “reading behavior”. This approach is representative of the vast majority of psycholinguistic studies of reading, such as the ones critically reviewed in the introduction. As the authors claimed, such approach allowed them to build a “well-controlled” study, unique in this area of hypertext reading. In this regard, the methods used are strong and accordingly the results based on the eye-movements indicators are reliable. However, the use of only word level indicators severely limits the scope of the studies, for at least two reasons.**

We agree that the design of our experiments occlude many claims being made about the nature of comprehension and navigation. We have amended the introduction and discussion of the manuscript to make the aims of this research clearer (i.e. the effect of navigation and skim reading within hypertext on lexical processing at the word level). We also discuss the need for further research into the important and linked areas of psycholinguistics and Web science that are noted by the reviewer. We reiterate that these experiments add to our ability to research these areas, as we have established effects at the level of lexical access. These experiments build on previous research we have conducted investigating the effects of hyperlinks on lexical access. We hope the foundations provided in these papers allow for the kinds of further research the reviewer discusses but are out of the scope of the current manuscript.  **Before elaborating on those, I should remark that there are alternative eye-movements indicators beyond word level ones, which allow to capture strategic reading behaviors, such as the ones we may expect to arose during hypertext reading. Among those, there are paragraph level measures, sequence indicators… Those have been used both in text reading research (e.g. Hyönä et al., 2000, JEdP) as well as in hypertext reading (e.g. Dugan & Paine, 2009, JEP; Salmerón et al., 2017, JCAL).**

While we do include passage reading times for Experiment 1 and 2, we do not provide other paragraph level measures. As the reviewer notes, we did not manipulate the goal of navigation, thus limiting the informational capacity of these measures. Our focus on word level measures reflects our aim to investigate the effects of introducing a Web-like environment on lexical access. **Regarding the problems associated to deciding to focus only on word level measures, the first one is that this methodological paradigm forces researchers to make strong design decisions to adjust the reading materials to maximize the experimental control. This is the same critique introduced in the manuscript when describing traditional sentence-long texts research literature on reading. Thus, a “well-controlled” study can become a reliable study but with poor construct validity. The most obvious limitation here is the claim that Experiment 1 was about hypertext reading, while no navigation was allowed. To me, such study corresponded clearly to the reading literature of text signaling devices (e.g. work by Lorch and Lemarié), but not hypertext, where navigation must be present.**

We have provided further detail in the introductions (lines 77-81, 205-209 and lines 539-545), which discusses the building up of our knowledge and understanding of reading behaviour on the Web at the word level. Experiment 1 allows us to establish a baseline of how readers read static web pages, for comprehension and when skim reading. This allowed us to make comparisons between these results and Experiment 2 in order to establish the unique contribution of navigation to reading, and this is reflected in the differential frequency and task type interactions we find across the Experiments.

**In Experiment 2, the limits of this approach were evident when authors decided that the task will be finished once participants have read ten pages. From the perspective of the participant as reading/student, I struggle to understand what would mean to me to “read a hypertext until you have reached 10 pages” (I assume this was told to the participants, although it was not clear to me what was exactly told).**

Our experiments allowed us to capture reading behaviour and lexical access within a task akin to navigating webpages. We have provided further clarity with regard to the exact instructions participants were provided with during Experiment 2 in the method section of Experiment 2. For eye movement studies the experimenter is present in the room running the equipment. They experimenter can see the content on the screen being viewed by the participant. The participant is instructed to read and navigate as they usually would in a Wikipedia environment and after 10 unique passages are read by the participant, the experimenter stops the data collection and starts up the next starting passage. If a participant uses the back button, the experimenter does not count this page twice. This way the participant has to do very little other than read the passages and navigate, resulting in natural reading behaviour. Note that reading web pages for a while without a clear target whilst clicking on the most interesting links and “taking it from there” will be far from atypical behaviour especially within a Wikipedia environment. At least anecdotally, many of us have acknowledged to regularly do this. Often the initial reason to go onto Wikipedia is practical but it starts an open-ended exploration.

**In the same line, in Experiment 2 pages were constructed to be independent from each other, if only because the comprehension questions only targeted information stated in a single page, and the participants are not given any particular reading goal emphasizing the need to strategically navigate the hypertext. This is a clever decision when you want to focus on word (or page for the matter) as an item, but it nevertheless makes navigation irrelevant in this context. Without any goal to guide her navigation, what is the participant meant to do? The two theoretical models discussed in the conclusion, the information foraging theory and the satisfying heuristic, start by providing participants with a specific goal (e.g. to find information, to write a summary, etc). Navigation takes place to efficiently achieve such goal.**

The goal for the participant was simply to navigate to anything they would like to read about as we did not want a separate task to interfere with the goal of viewing normal reading behaviour. As we previously stated, we accept this limitation of the study, as our study is concerned with word-level processing differences. This is now addressed in the conclusion. We note that our results are notable for the interesting effect of navigation, even when the task is not critical to any informational goal beyond correctly answering comprehension questions, and that further research should investigate how different goals affects reading behaviour beyond our established baseline (lines 1013-1026).  **On a different note, I have several concerns regarding the type of comprehension questions used in both studies:
-In experiment 1, comprehension scores were really high, suggesting that there was a floor effect. This limits the chances to capture differences between conditions.**

We acknowledge in the paper that the goal of comprehension questions is as a task validity check (i.e. to show participants were reading as they normally would, and were understanding the text), which is typical of the eye movements in reading literature (line 328-332). Often very easy questions are used for this goal. As such, we usually expect to achieve near ceiling effects for this measure. This is explored in the discussion (line 1040-1045). It is important to note that for this reason comprehension questions in eye movement during reading studies are often answered with an accuracy that is at or near ceiling level and that comprehension accuracy is typically reported but not necessarily analysed as function of experimental conditions in these experiments.

**-In both studies, questions only asked information presented in a single page. This represents a rather limited view of text comprehension, as it ignores integration processes that take place when students relate information across pages.**

As previously mentioned, these integration processes are very interesting, and requires further investigation to provide a fuller understanding of text comprehension when reading on the Web, and this is now discussed in the discussion.

**-In both studies, if I got it right, comprehension questions were provided immediately after leaving a page, instead of being provided at the end of the reading session. Such combination emphasized the idea that participants had to focus on the single pages, and that there was no need to strategically navigate across the pages. Why not just giving the comprehension questions after the reading session?**

Comprehension questions during eye movement and reading studies are typical of this research area, and are provided during the task in order to ensure participants are maintaining their attention throughout and ensure our reading task is valid and reflective of normal reading behaviour. This is now referred to in the discussion (lines 1040-1045). An alternative method would be useful for investigating different research questions to the one considered in this paper. Further research exploring information integration across multiple pages would benefit from having questions at the end where they cannot disrupt the integration process, but for the current studies we just needed to make sure the reader was reading the content so we could draw conclusions about their reading behaviour at a word level.

**-In experiment 2 I find it hard to understand how comprehension could be assessed, and compared across participants, given that participants could read a different set of materials in each condition, and questions were aligned to specific pages. Is there any evidence that the difficulty of the comprehension questions was similar across pages?**

We provide more detail to demonstrate the nature of the comprehension questions in the method section of Experiment 1, explaining they always ask relatively basic information about the passage. As we have not made claims about the comprehension results, further investigation of comprehension results, or a difficulty comparison would not be appropriate for this manuscript (as discussed on line 1030-1035). Additional material to display the nature of the comprehension questions can be found here: <https://drive.google.com/drive/folders/1MCg0CPjifdEttkCjqZXyqeKLimmzZMX-?usp=sharing>

**All in all, I have confronted feelings on the manuscript. On the one hand, I really value the effort to build well controlled studies in a complex scenario such as hypertext reading. The approach taken in these studies is not only excellent but also necessary in an area in need for well controlled studies. But in this particular attempt, I am afraid that authors have gone too far by overvaluing experimental control over construct validity.

Minor point
Table 1. (190 or (19)?**

This has been amended.  **Reviewer #3: In the manuscript entitled "The Impact of Skim Reading and Navigation when Reading Hyperlinks on the Web" the authors report two experiments using eye-tracking measures to study effects of hyperlinks on reading behavior. The authors describe a complete within-subjects design, in which they experimentally manipulated three factors: reading for comprehension or skim reading (i.e., task), target words marked as hyperlinks or not, and target words of high frequency or of low frequency. In Exp. 1 participants had to read only, in Exp. 2 participants could navigate through an entire hypertext-environment by clicking on the words marked as hyperlinks. The authors observed in Exp. 1 a word frequency effect (i.e., a difference between words of low and high frequency) to be present only during skim reading for words marked as hyperlinks. This outcome was interpreted as showing that during skim reading only hyperlinked-words were fully lexically processed. In contrast, in Exp. 2 when the hyperlinked words were clickable, the authors observed under both task conditions that only words marked as hyperlinks were fully lexically processed.

The manuscript is generally well written and adds some additional insights in the effects of hyperlinks on reading, strongly building upon previous research conducted by the authors.

However, I do have some major concerns the authors might thoroughly address before publication. Most of these concerns equally address both experiments, although I might only refer to specific examples of the first or the second experiment.

1) I missed a more detailed description of the actual task materials used respectively had some problems in understanding the design of the task materials based on the description provided by the authors (this holds true for Exp. 1 and 2).**

We have added details based on the reviewers recommendations across the methods of Experiment 1 and 2. We also signal readers towards a technical account of the clicking environment provided by Fitzsimmons which provides a much more detailed account than is possible within this paper, this methodology paper is cited within the manuscript (<https://eprints.soton.ac.uk/438159/1/A_Novel_Methodology_for_Creating_an_Interactive_Non_Linear_Space_in_an_Eye_Tracking_Environment.pdf>) and more information can be found in Fitzsimmons, G., Weal, M. J., & Drieghe, D. (2019). The impact of hyperlinks on reading text. PloS one, 14(2): e0210900, where the stimuli are adapted from. **For Exp. 1&2: What were the topics of the 40 articles and how long were the articles? Based on which ratio were the target words in the sentences selected? Were the target words at a specific position within the sentence or equally distributed across the sentences? How long were the sentences? Were the target words of a specific category? Were the sentences of a specific topic?**

We have now provided more detail about the nature of the target words and their distribution across the page in the method section of Experiment 1. More information can also be found in Fitzsimmons, G., Weal, M. J., & Drieghe, D. (2019). The impact of hyperlinks on reading text. PloS one, 14(2): e0210900., where the stimuli are adapted from.  **If I understand it correctly, the experimental manipulations in Exp. 1 were done in specific sentences that were then inserted into pre-existing Wikipedia-articles. How was the topical congruency between the inserted sentences and the rest of the article ensured? Did this procedure result in realistic Wikipedia-like articles?**

Target sentences were adapted from Wikipedia to include a high/low frequency manipulation and were designed to provide realistic Wikipedia-like articles to participants. An additional example of the stimuli and comprehension questions can be found here: <https://drive.google.com/drive/folders/1MCg0CPjifdEttkCjqZXyqeKLimmzZMX-?usp=sharing>. As with Fitzsimmons et al. (2019), the stimuli were designed to be natural and semantically consistent with the previous text and Wikipedia articles, and we now discuss this point in the Materials and Design section of Experiment 1.

**As the authors note, pre-existing hyperlinks in the articles were retained (the authors might specify the number of those 'other' hyperlinks). If I understand that correctly, there was an unbalance in the total number of hyperlinks in the articles based on whether the articles were used for the hyperlink-condition or the no-hyperlink-condition, that is in the hyperlink-condition there were more hyperlinks in the article as compared to the no-hyperlink condition. Potentially, the presence of additional hyperlinks in the no-hyperlink-condition could have severely influenced the processing of the non-hyperlinked target word, that is, at least partly explain the reduced lexical processing thereof during skim reading as participants might have predominantly focused on the remaining hyperlinks.**

We omitted some details in the original manuscript for the sake of brevity. On the recommendation of the reviewer and to provide greater clarity, we have provided much more detail regarding the number of links and greater control we had of these. We balanced the number of links and rotated which were linked/unlinked. As such, we do not believe this would have affected our results. This information is now provided in the Experiment 1 method.

**Besides, Figure 1 and Figure 5 that should show examples of the task materials are missing, hindering a correct understanding and discussion of the materials.**

Links to these figures are now provided in the manuscript **2) I suspect (but maybe I am wrong) in Exp. 1 the manipulation of hyperlink present/not present and word frequency was done article-wise. If this interpretation is correct than that’s an additional difference to Exp. 2 that should be discussed as in Exp. 2 both hyperlinked and non-hyperlinked target words had to be present within the same article to allow for navigation. It would be helpful if the authors thoroughly revised these sections of the manuscript. Or to put it in other words: did each article contain each manipulation (i.e. equal amounts of low/high frequency words, hyperlinks/no hyperlinks) or were the conditions manipulated per article (i.e., e.g., 5 articles skim reading and low frequency target words and no hyperlinks)? Were the experimental manipulations bound to a specific article or were they equally distributed across articles?**

We have provided more detail regarding the linked/unlinked manipulation and the randomisation of trials within each task (comprehension/skim reading) to clarify these points in the method section of Experiment 1. As such, it is explained, in Experiment 1, that presence of links was manipulated within trials, across the four sentences embedded within each trial, as opposed to between trials.

**3) The fixed order of first, reading for comprehension, and second, skim reading in both experiments is problematic, as it confounds the manipulation of reading task with potential sequential effects (like tiredness, boredom, or the fact that participants generally might getting used to the overall task). Therefore, it is questionable whether the observed effects on skim reading could really be attributed to skim reading or might be (at least partly) due to confounding factors. The authors clearly should state this as a limitation of their study that has to be discussed more thoroughly in the general discussion section. Maybe in an additional analysis, the authors could split the reading for comprehension phase into two parts and see whether in the second part the reading behavior has already become more skim-reading like. If this is not the case, this outcome might be used as an argument why the fixed sequence might not be problematic. Besides, the argumentation of the authors for the fixed order could be also reversed. Potentially, if the participants start reading for comprehension at the beginning they might continue to do so throughout the entire experiment (especially due to the repeated knowledge tests).**

We considered this point whilst constructing these experiments, and were motivated by some piloting of the task. Readers in this pilot study reported finding it hard to switch to reading for comprehension after they had been skim reading for a while.

On the advice of the reviewer, we ran additional LMMs with trial number added as a predictor and compared the fit of these models with the ones used in the manuscript. We found no significant effect of trial number in any of our measures across all experiments (*t*s <1.96). We also found models including the trial number did not significantly contribute towards explaining variance within our data. Comparison of model with time added compared to model used in paper – Experiment 1 - Go-past time - *X^2^* (8) = 9.72, *p* = .285, Single fixation duration - *X^2^* (8) = 10.55, *p* = .228, Experiment 2 first fixation duration – *X^2^* (6) = 5.29, *p* = .507, gaze duration - *X^2^* (8) = 10.55, *p* = .229, go-past time - *X^2^* (8) = 11.75, *p* = .163, single fixation duration - *X^2^* (8) = 11.71, *p* = .165.

The one exception to this, however, was total reading time in Experiment 2 (*X^2^* (8) = 1435, *p* < .001), where including trial did significantly improve the fit of the model. It did not, however, have a significant effect on our results (main effect of time – *t* = 1.66, and t<1.96 for all two, three and four-way interactions with other variables) and did not affect the pattern of results (i.e. we still found a significant effect of frequency (*t =* 2.11), word type (*t =* 3.60), and task type (*t =* 3.33)). As such, we do not believe that this aspect of the task influenced our overall pattern of results, and that task order did not influence our results in any meaningful way.

**The authors should report the entire reading times for the two tasks in Exp. 1 and might analyze reading times for differences between skim reading and reading for comprehension.**

We now report passage and analyse passage reading time in Experiment 1 as well, to further validate our skim reading manipulation and ordering of the blocks of trials. **4) Were the knowledge tests specifically related to the target words/sentences or rather to the rest of the article?**

We have provided more detail on the advice of multiple reviewers regarding the comprehension questions. We clarify they ask for detail regarding the entire article, not just to the target word in the procedure section of Experiment 1’s method. **5) What exactly were the task instructions for the two tasks? Were the participants informed about the following knowledge questions? How (i.e., did they get a global task of remembering everything or specific aspects of the texts only?)?**

We have provided further information about the instructions participants were given in the method of Experiment 1 (which is also relevant to Experiment 2). Participants knew there were going to be comprehension questions for all passages in Experiment 1, and for some in Experiment 2 and that the questions would pertain to the information read in the previous text only. **6) I would be interested to hear some arguments of the authors why they decided to use linear mixed models for their statistical analyses. Based on the clear 3-factorial task design and the research question I in a way would have expected classical repeated-measures ANOVAs. What is the addon of using LMM in this scenario? Besides, I would be curious whether repeated-measures ANOVAs would show up identical results.**

On the advice of the editor, we have not addressed this comment. LMMs provide a method of data analysis that deals with missing data much more robustly than traditional ANOVAs. Missing data is a particular issue in eye movement studies (due to word skipping), and even more common in a study where participants can click to end a trial before reading the entire text. They also take into account the random effects of participant and stimuli, avoiding the need for F1 and F2 type analysis, as is common when using ANOVA.  **7) I miss a thorough introduction and motivation of the eye-tracking measures used in the two studies (also backed up with more literature). For example, in the introduction the authors state skim reading is characterized by fewer fixations on words, but this measure is not used by the authors to disentangle skim reading from thorough reading. Reporting such additional, established eye-tracking measures might be valuable as a manipulation-check of the reading tasks. Besides, for me it did not become fully clear why the authors did not use the same measures in both experiments and why they only focus on measures with respect to the target words.**

We have now provided passage reading time for both Experiment 1 and 2 as a global measure, which further validates our skim reading manipulation. As we state in the manuscript, the choice of different analyses was inherent on the amount of single fixations made on a word. This occurred in 94% of cases in Experiment 1, meaning measures like total reading time and go past reading time have very limited additional informational capacity (e.g. total time will usually be the same as single fixation duration as they usually only made one fixation, go-past time would only be different in the <6% of cases where readers actually made a regression back into the text). This is not the case in Experiment 2, where single fixations on the target word were much less common, vastly increasing the informational capacity of the other eye movement measures. We could mention these measures in both parts of the paper, but have chosen not to do so as they provide no additional insight into the findings in Experiment 1.

**Especially in the non-hyperlink condition, the target words were opaque to the participants, so it is kind of trivial that participants fixated hyperlinked target words differently than the other words (this in a way relates to my question, what was specific about the target words).**

We believe our findings for the target words in terms of frequency effects as a function of whether the target word was a hyperlink or not and whether the task was skim reading or reading for comprehension to be far from trivial. They provide novel evidence regarding the processing of hypertext at the word level.

**As the authors state, they were interested in how hyperlinks and hyperlink-selection affects reading behavior I would have expected to hear more about more global eye-tracking measures on the sentence or text level (like e.g., total number of fixations).**

Concerning the request for more global eye-tracking measures, we had great experimental control over the target words, in terms of frequency and word length, making analysis of fixations on a single word well justified. As we wanted to create an as Wikipedia-like environment as possible, we did not control sentence length (or indeed passage length) meaning any comparisons of measures like these would be influenced by the lack of experimental control we have over them. We agree this is of particular interest to the research area. We, however, believe this to be outside of the scope of our paper, which focuses on word level effects, but we do discuss this as a point of further research in the discussion (line 1013-1018).  **Is the measure 'go-past time' equivalent to the dwell time?**

We clarify go past time in the results section of Experiment 2, explaining it accounts for any time spent fixating on the target and any fixations made back into the text before moving past the word. As such, it is the time taken from fixating on the target to going past it and onto the next work to the right.  **8) The transition from Exp. 1 to Exp. 2 might be kind of optimized. Based on the discussion of Exp. 1 I would have expected the story would go to look at the effects of highlighted words in general (which the authors already addressed in previous research, so this research might be referenced here). In a way, I am missing a more thorough motivation for conducting Exp. 2. Besides, as the authors started the manuscript by describing classical reading research and the artificial task materials used in these studies, one might state that the task materials the authors used in the current study still seem kind of artificially (which is legitime in doing good research, yet, the argument of ecological validity implicitly stated at the beginning of the manuscript might still only partly be redeemed. For example, in Exp. 2 the single Wikipedia-articles seem to consist of only some sentences (and in addition no other elements, like pictures), therefore the question arises how realistic the material was actually percieved by the participants.**

On the advice of this reviewer and reviewer 2, we now provide a stronger link between the two studies, detailing how they are building up our understanding of reading on the Web. This detail is provided in the introduction to Experiment 2, and in our discussion of limitations in the general discussion. We endeavoured to make the stimuli as similar to Wikipedia as possible while retaining experimental control. As the first study exploring this type of reading using eye-tracking methodology we wanted to make it as controlled as possible to explore only reading for this task. We did not include images because this would add variance to the passages and could impact the analysis of reading behaviour. If someone was to begin reading and glance to the image, it would make reading measures difficult to interpret. Instead, every article included the Wikipedia header and left-hand sidebar, the title of the page was formatted as Wikipedia is formatted. The content was mostly taken directly from Wikipedia so the style of the content was consistent, only the experimental sentences were manipulated to add target words. Future research could explore the impact of images etc on Wikipedia-like article, but that would be outside of the scope for the current article.  **9) The interpretation that target words were differently lexically processed seems to be a quite strong conclusion given that no direct measure of (or probe for) lexical processing is provided.**

The frequency effect has traditionally been taken as the most reliable of lexical access, hence it was used in this study to support our claims regarding lexical access. This is extremely common within the eye movement and reading literature and has been used as an indices of lexical access in many different research areas. We now discuss this in the introduction, referring to visual search of text and proofreading studies which support this theoretical assertion (line 210-218).  **10) Were the comprehension scores in Exp. 2 significantly different for the two reading tasks?**

We have not analysed the comprehension scores. This is an issue also addressed in our response to reviewer 2, where we state that the main aim of the article is to explore lexical access during reading of a Web environment. Comprehension scores are designed to ensure participants read as they normally would and understand the text. As they are validity check, we refrain from analysing them as a dependent variable in our study. There were also other factors (e.g. the fact that readers answered different questions dependent on the web pages they were reading in Experiment 2) that would stand in the way of interpreting the accuracy to the comprehension questions in too much detail.  **Some minor comments:

• There are some typos (e.g., Table 1 a 'zero' instead of a closing bracket) and some grammatical inconsistencies (e.g., sometimes use of simple present instead of past tense when describing the study).**

We have proofread the manuscript to remove these typos, and adjusted the wording of the method sections on the advice of the reviewer.  **• The result section of Exp. 2 might be more closely structured as that of Exp1 (especially with respect to sub-headings).**

The sub-headings of the results section of Experiment 2 have been adjusted to match those of Experiment 1 **• Exp. 1 states that participants did not take part in Exp. 2. However, a comparable information is missing in Exp. 2. Does this mean Exp. 2 was actually conducted before Exp. 1?**

We now further clarify that those who took part in Experiment 2 did not take part in Experiment 1 in the participants section of the method, just as those who took part in Experiment 1 did not take part in Experiment 2.  **• Exp. 2 how many target words (i.e., trials) on total get into the statistical analysis (i.e., how many potential target words were excluded from analysis due to the criterion stated l. 521? Could this result in an unbalanced amount of target words for the hyperlinked/non-hyperlinked task conditions?)?**

The number of target words in each analysis is provided in response to a similar comment from reviewer 1. While there is imbalance within the design, LMMs handle missing data much better than traditional ANOVA .

**• Exp. 2: How many hyperlinks were actually clicked? Was there a different clicking-pattern between the task conditions (i.e., skim reading or reading for comprehension)?**

We did not analyse clicking behaviour because, as reviewer 2 has stated, these were not critical to the task the participant completed, and are thus inappropriate for analysis within this study. We mention this point in our consideration of further research in the general discussion.  **• Figures: Maybe the figures could be replotted in a more condensed way, e.g. with the data for the two reading tasks in the same plot (e.g., using color-coding). This might visually help in comparing the outcomes of the experimental conditions and would reduce the (rather high) total number of separate plots.**

We considered this design change but found the figures were often harder to interpret, due to overlap between data points and error bars.
