# Supplementary material for: The impact of skim reading and navigation when reading hyperlinks on the web
Source: PLoS One. 2020 Sep 17;15(9):e0239134. doi: 10.1371/journal.pone.0239134 (PMC7497986; doi:10.1371/journal.pone.0239134)
Supplement: Attachment — Submitted filename: Response to Reviewers.docx [file pone.0239134.s002.docx]

Thank you for submitting your manuscript to PLOS ONE. The reviewers were generally pleased with the changes implemented in your revised manuscript; however, there are some relatively minor residual concerns that need to be addressed before it is accepted for publication. These include issues with data reporting and clarity of the Introduction.

**We have now made the suggested changes, particularly regarding data reporting and the introduction.**

Reviewer #1: This manuscript reports two experiments examining the impact of hyperlinks on eye movement behaviour during reading. I was reviewer 1 previously.

My main concern with the previous manuscript was that there appeared to be some errors in the reporting of the results. I am satisfied that this was simply a labelling error, and everything seems to now match up. I have only some minor comments about this updated manuscript:

Abstract lines 26-28 – This is a little ambiguous – it could be interpreted as that readers only lexically process linked words when skim reading and not when reading for comprehension. There is a similar issue in line 434.

**We have altered the wording of these two sentences to remove the ambiguity over the findings between skim reading and reading for comprehension.**

Lines 359-361 – You report skipping probabilities here, but these don’t seem to match with what is reported in table 1.

**We have removed this incorrect information from the manuscript, the reader is now referred to table one, where it can be seen that skipping rates are significantly higher for high frequency words than low frequency.**

I presume the data in Figure 3 has been log-transformed (as it has for the linear mixed effects models). This should be mentioned (perhaps on the label for the Y axis) as this is fairly unusual.

**The go-past time measures were log transformed in this experiment, and Figure 3 displays the results from this LMM. We now also mention that these data are log transformed in the caption for this figure to make this clear.**

Reviewer #3: In the revised version of the manuscript entitled "The Impact of Skim Reading and Navigation when Reading Hyperlinks on the Web" the authors addressed most of my previous concerns satisfactorily.

Yet I still do have some (mostly minor) concerns.

The authors now provide a more detailed description of the task materials. Still I do think an example of the task materials used should be shown directly in the manuscript as a figure. It would be nice to directly highlight in this exemplary task material the additionally inserted sentences and hyperlinks (i.e., making the experimental manipulation directly visible). Note that I am rather asking for a schematic depiction of the task materials showing the experimental manipulation at a glance, not necessarily the real materials with the Wikipedia logo. The real task material still could be provided via a hyperlink, yet I am kind of skeptical whether a link to Google drive is a very sustainable solution (i.e., can the authors ensure that the link is working in five or ten years?). Generally, I think for readers it is preferable to have all relevant information on a study in the manuscript at hand and not only via references or links to other sources.

**We have added an additional schematic figure to convey the stimuli, as well as the high/low frequency and linked/unlinked manipulations (figure 1).**

As the authors now clearly limited the scope of their research question to the lexical processing of hyperlinks at the word level, I suggest also revising the abstract accordingly. In its current form, the initial sentences still raise the expectation that the authors' study deal with the effects of hyperlinks on text comprehension and reading behavior. Yet, the authors specifically only focus on the word level (and only on specific hyperlinked words). This is a valid approach, but it should be made very clear early in time. Also, with respect to the last sentence of the abstract one might wonder whether this conclusion is not too broad (and strong) given the specific focus of the experiments.

**We have altered the wording of the of the abstract (particularly the first and last sentence) to show the reduced scope of our findings to lexical processing of single words.**

The authors might also think about revising the beginning of the introduction. Starting with differentiating reading online text versus reading in reading experiments is kind of misleading as the authors' study does not a direct comparison of these two reading tasks. Also, the different reading strategies are induced via instruction, that is, skim reading could also be instructed in classical reading experiments. Besides, offlinen reading might be considered also to be very different from reading in typical reading experiments. As noted for the abstract, I would suggest being clear about the specific focus of the authors' study early in time to avoid raising false expectations among readers.

**We have amended our discussion of the focus of the study to be more specific, to avoid any ‘false expectations’.**

l. 57: A short definition of skim reading might be helpful for the general reader here.

**A short definition of skim reading is now provided when it is first mentioned in the Introduction.**

The use of the general term 'reading behavior' (e.g., used l. 191) might be considered as being too broad for the specific focus of the authors' study. The authors might clarify (i.e., define) early in time what is meant by reading behavior in the context of the current study.

**While we maintain the use of reading behaviour, as that is what our participants were engaged in by the skim/comprehension manipulation, we have included more references throughout to its effect on lexical processing, as it is what our dependent variables represent. We have made minor amendments to make this distinction clearer within the text.**

l.299: The authors might provide a reference for their outlier definitions of fixation durations (i.e., why merging fixations shorter 80 ms, why not only those shorter 50 ms; why removing fixations longer 800 ms).

**We now provide references regarding data cleaning to evidence our procedures for this were the standard with eye movement and reading studies.**
